# Experiences of medical interns of emotionally charged workplace encounters: A qualitative exploration of their rural rotation

**DOI:** 10.4102/phcfm.v18i1.5435

**Published:** 2026-08-12

**Authors:** Madeleine Muller, Oladele V. Adeniyi, Susan van Schalkwyk

**Affiliations:** 1 Department of Family Medicine and Rural Health, Faculty of Medicine and Health Sciences, Walter Sisulu University, East London, South Africa; 2 Department of Family Medicine, Cecilia Makiwane Hospital, East London, South Africa; 3 Department of Family Medicine and Emergency Medicine, Cecilia Makiwane Hospital, East London, South Africa; 4 Department of Health Professions Education, Faculty of Medicine and Health Sciences, Stellenbosch University, Cape Town, South Africa

**Keywords:** emotional competence, emotional regulation, junior doctors, medical interns, resource-restrained hospitals, rural hospitals

## Abstract

**Background:**

Junior doctors experience high-pressure emotional interactions, often with limited support during their rural hospital rotation. Little is known about the effect of such encounters on the emotions and behaviour of clinicians.

**Aim:**

This study explores the experiences of interns during emotionally charged encounters in the workplace with clinical supervisors, colleagues and patients.

**Setting:**

The study was conducted in a one regional and three rural district hospitals.

**Methods:**

A qualitative exploratory study was conducted using semi-structured interviews with 12 interns undergoing training in the Eastern Cape, South Africa, between May 2024 and August 2024. A purposive sample of second-year medical interns who had completed a 2-month rotation in a rural district hospital setting was interviewed. Data were coded and subsequently analysed thematically.

**Results:**

Three themes emerged: ‘being a doctor in an emotionally charged clinical environment’, ‘the emotionally competent doctor’ and ‘the emotional support needs of junior doctors’. Emotionally charged clinical encounters elicited strong, distressing emotions in interns, including anxiety, frustration, and feelings of helplessness, which affected their well-being, service delivery, and clinical performance. Unregulated emotions resulted in counterproductive behaviours, such as abruptness, avoidance, or reluctance to seek guidance. Participants expressed a need for structured emotional competence (EC) training and additional emotional support.

**Conclusion:**

Junior doctors face emotionally charged encounters in their clinical practice. Unregulated emotions may drive counterproductive behaviours, affecting service delivery and patient care. Effective educational activities need to be developed to support the development of EC in undergraduate medical education. A programme targeting the emotional well-being of doctors, especially junior doctors, could provide additional support.

**Contribution:**

Identifies a need for EC training of doctors.

## Introduction

Strong emotions affect behaviour.^1,2^ Clinicians are exposed to highly charged clinical environments, especially interpersonal and professional conflicts, which have been linked to high rates of burnout and emotional exhaustion,^3^ particularly in rural or resource-strained settings.^4^ Clinicians need to be mindful of their own emotions and those of their patients and colleagues during highly stressful, emotionally laden encounters and respond appropriately. This requires specific skills.^5,6,7^ The ability to manage emotions is captured under terms such as *soft skills, socio-emotive* and *communication skills*,^5^ and is found in frameworks such as emotional intelligence and emotional maturity.^8,9,10^ *Emotional competence* (EC), proposed by Mikolajczak^11^ and expanded on by Brasseur et al.,^12^ is a term that draws these concepts together. Emotional competence includes three levels of competence: emotional insight (knowledge of how emotions impact behaviour), emotional awareness (ability to reflect on emotions), and emotional regulation (ability to regulate emotions and related behaviour).^11,12^

In South Africa, junior doctors gain their first experience taking responsibility for patient care in a rural or resource-constrained setting during their second-year internship, when they spend 2 months at a district hospital. The interns are incorporated into the workforce, often with less supervision than in large regional hospitals, and may be exposed to high-stress environments with minimal support. As newly qualified clinicians, many are still in their twenties or early thirties and must manage challenging encounters with patients, family members, or colleagues in challenging, resource-constrained settings.

The ability to manage emotions has significance in the workplace. It is well known that high-pressure environments contribute to burnout and mental illness.^3,4^ It is also known that patients evaluate their physicians based mainly on their socio-emotional behaviour, which affects patient trust, self-disclosure, and satisfaction.^13^ In addition, socio-emotive skills have been shown to reduce practitioners’ mistakes and ultimately improve healthcare outcomes.^5^ What is not yet understood is the impact of emotionally laden, high-stress encounters on the emotions and behaviours of doctors in rural, resource-restrained settings. The research question in this study asks how medical interns experience emotionally laden encounters in the workplace and how these experiences affect their behaviour in clinical encounters.

## Research methods and design

### Study design

An explorative, descriptive qualitative study was conducted at a regional hospital in the Eastern Cape, South Africa. A qualitative study design enables the researcher to explore the lived experiences of the study participants.^14^

### Setting

After completion of medical school in South Africa, graduates have to complete a 2-year internship doing block rotations in an academic hospital setting. During their second year of internship, the interns rotate through 6 months of family medicine, which includes clinical exposure in a rural district or regional hospital setting. This study was conducted at an academic hospital in Buffalo City Metro and included experiences from three districts in the Eastern Cape of South Africa. Interns are placed at one of four rural district or regional hospitals for 2 months during their family medicine rotation. These hospitals are based in under-resourced rural areas, with most day-to-day supervision being provided by medical officers. Interns are incorporated into the workforce and are expected to provide clinical services, including seeing patients in the emergency department, maternity wards and adult and paediatric wards. Although clinical supervision is available when needed, interns often provide care independently, especially after hours. After their rotation, interns return to the academic hospital to complete their block rotations.

### Study population and sample size

The study population comprised 24 second-year interns who rotated through the family medicine department from January 2024 to June 2024 and had completed 2 months’ service in one of the four rural district hospitals at the time of data collection. All 24 were invited, and 12 interns volunteered to participate in the semi-structured interviews. We considered information power, rather than saturation, to determine the number of participants who would provide adequate information in a qualitative study.^15^ Patton^14^ asserts that including between 4 and 15 participants in a qualitative study ensures diversity while keeping the amount of data manageable. As the aim of the study was to do an in-depth analysis of narratives from a very select group (interns in a rural district hospital setting), a sample of 12 participants was considered sufficient for the study.

### Data collection

First author and trained interviewer (Madeleine Muller) conducted 12 semi-structured individual interviews with prompts over 2 months, from May 2024 to August 2024. An interview schedule was developed, guided by the research question, to explore a recent emotionally charged interaction that the participant had encountered. After an introduction to the study and questions about the intern’s district hospital rotation (where they were placed and their overall impression), the following opening question was asked: *‘I would like us to explore one or two events during your district hospital rotation. It is not unusual to be exposed to emotionally charged situations. This can be a difficult patient encounter or an encounter with a colleague or supervisor. You are welcome to choose any scenario you are comfortable sharing, and it does not necessarily have to be the most challenging one. We want to explore what you felt during this encounter’*. Interview questions elicited details of the encounter and included probing questions to encourage deeper exploration of the emotions and behaviours experienced by the participant and others in the workplace.

The interviewer elicited rich, in-depth descriptions by adopting an open-ended interview technique.^16^ The same interviewer conducted all the interviews to maintain consistency throughout the study period. The interviewer made notes based on direct observation of the participants’ feelings, thoughts, and intentions as expressed in body language, which could not be captured on the recording. The interviews lasted between 39 min and 68 min and were recorded on a laptop using Otter software,^17^ which also transcribed the audio data in real time. All interviews were conducted in English, the medium of communication among the participants, and took place at the family medicine offices during working hours at the hospital where the interns were placed.

### Data analysis

The voice-to-text transcriptions were checked for accuracy within 24 h of each interview. Field notes were reviewed for additional information. The data were coded using Atlas.ti.^18^ Reflexive thematic analysis was employed to identify, analyse, and report on patterns in the data, using the six phases outlined by Braun and Clarke.^19^ After familiarisation with the data, initial codes were generated to organise the data into meaningful clusters.^20^ Coding was data-driven, using an interpretative approach grounded in the participants’ verbal reports. Each segment of data was coded accordingly, with the co-author (S.V.S.) coding the first interview in parallel with the first author (M.M.) to ensure that coding was meaningful and logical.^14^ Through a thorough, iterative process of data engagement, several themes were identified to represent the data.^14,16^ This study captures the three themes that emerged most strongly in relation to the research questions.

### Trustworthiness

As detailed above, a range of measures was applied to ensure the study’s trustworthiness.^14,20^ These included steps to ensure credibility (triangulation of data analysis by involving two experienced researchers (M.M. and S.V.S.) to review the coding and thematic analysis), transferability (a detailed description of the research process, the setting and who was interviewed) and dependability (capturing the interviews in detail, including verbatim quotes).

To ensure confirmability, the authors had to continuously reflect on their own biases and positionality and be transparent about choices and underlying assumptions.^14,16^ The first author (M.M.) is a family physician and senior lecturer in the family medicine department at the academic hospital where the interns had been placed for their family medicine rotation. She provides mentoring and clinical support for interns in the family medicine outpatient department and HIV clinic. M.M. provided no direct supervision for the interns during their placement in the district hospitals, but was aware that interns had experienced challenges and high levels of stress during these rotations in the past, and was interested in how to better capacitate interns to manage these situations. M.M. reflected on her assumptions and beliefs prior to the study, particularly regarding the level of support and the impact of emotionally charged interactions on interns in rural, resource-restrained settings. A clear interview guide with open-ended questions during the interviews aimed to capture the impressions of the interns, and M.M. was aware during data collection and data analysis not to let her own preconceived ideas around emotions influence the information captured. This was the first time M.M. had conducted a qualitative study. As it formed part of a master’s in health professional education, M.M. had both extensive theoretical training and supervision from S.V.S. in all aspects of the study. M.M. conducted all the interviews and therefore deemed it necessary to reflect on her role in the research process and possible influence on the data. This included considering the researcher’s role as a departmental consultant. Although not responsible for the interns’ assessment, the interviewer (M.M.) recognised her role as a consultant within the department and the influence this might have on the interview process, which had to be moderated.

### Ethical considerations

Ethical approval was granted by Stellenbosch University’s Faculty of Medicine and Health Sciences’ Health Research Ethics Committee (Reference Number: S24/02/044). Permission to implement the study protocol was obtained from the Eastern Cape Department of Health and the hospital’s clinical governance. Each participant provided written informed consent prior to recruitment into the study. The study followed the Helsinki Declaration and Good Clinical Practice Guidelines.

## Results

### Characteristics of the participants

The final group represented a typical distribution of medical interns by race, sex, age, and undergraduate medical universities attended. To protect anonymity, detailed demographic information about this group is not provided. The sample comprised four women and eight men, with a median age of 26 years. They had completed their undergraduate studies at eight South African medical universities. Two participants had been part of the Nelson Mandela Fidel Castro programme, spending the first 5 years of their training in Cuba before completing their studies at a South African university.

From the thematic analysis, three themes emerged that captured the effects of emotionally charged clinical encounters on junior doctors in the workplace (Table 1). Theme 1, ‘Being a doctor in an emotionally charged clinical environment’, describes the effects of working in these environments. Theme 2, ‘The emotionally competent doctor’, concerns the EC skills that junior doctors already possess, and Theme 3, ‘Emotional support needs of junior doctors’, concerns the doctors’ perceptions of what they need to assist them with managing emotions in these high-stress environments.

**TABLE 1:** A summary of themes and sub-themes.

Themes	Sub-themes
Theme 1: Being a doctor in an emotionally charged clinical environment	Sub-theme 1.1: Experiencing emotions in the clinical environment Sub-theme 1.2: The influence of emotions on service delivery Sub-theme 1.3: The influence of emotions on work performance
Theme 2: The emotionally competent doctor	Sub-theme 2.1: Interns’ inherent emotional management skills Sub-theme 2.2: Specific skills for managing emotions in the workplace
Theme 3: The emotional support needs of junior doctors	Sub-theme 3.1: Sources of emotional support for medical interns Sub-theme 3.2: Workplace support for medical interns

#### Theme 1: Being a doctor in an emotionally charged clinical environment

Participants’ descriptions of emotionally charged interactions presented a vivid picture of how these events affected their well-being and performance in the clinical environment. Three sub-themes were identified:

**Sub-theme 1.1: Experiencing emotions in the clinical environment:** The participants disclosed that they were overwhelmed, fatigued, and stressed by emotionally charged workplace interactions. The most common distressing emotion was a sense of powerlessness when presuming they had failed a patient or disappointed a senior doctor. This sense of powerlessness also encompassed inadequacy, embarrassment, and despair, as expressed in the following extract:

‘It was … this sense of, like, guilt or embarrassment, or shame that I couldn’t do more for this patient. And then also … I just felt like hurt, like sadness …’ (P04)

Participants often had to deal with difficult and tragic events, which strongly affected them:

‘And one night … two kids died. And the mothers were, obviously hysterical, and I feel like that night was very tough … I’ve actually never cried about medicine before, and that was the first night. I feel like that was just a very emotional night. Obviously, you know, two kids dying … and seeing the mothers.’ (P03)

Another participant highlighted their sensitivity to the suffering of others:

‘I am sensitive. I’m very sensitive. I don’t like suffering in people, I don’t like to suffer myself.’ (P11)

A common challenging interaction, as described by the participants, was conflict with patients’ angry family members, who might threaten to report the doctor, question their competency, or insult them in anger and irritation. The following quote refers:

‘It became like very personal, and then that became very difficult for me to … navigate, and it’s, like, depressing, because I mean, the whole reason why I came into medicine was to help people, and these persons are directly telling me that I’m a bad person, and I’m not willing to help someone in need. So it was very difficult for me.’ (P04)

When the participants were criticised, they would experience anxiety, which would be triggered whenever they encountered similar clinical scenarios, even as they strove to forget the incident. One intern described the emotion experienced on the day after the death of two children during the night, while she was on call, as follows:

‘… just knowing that a child can crash on you, always having that feeling, that looming feeling …’ (P08)

**Sub-theme 1.2: The influence of emotions on service delivery:** Participants reported how emotions influenced their behaviour. Sometimes, this behaviour affected patient care. They noted how some incidents triggered strong emotions that changed how they, or senior colleagues, behaved towards patients, potentially for several hours after the incident. The influence of their emotions could be discerned in changes in their demeanour, which might become more abrupt, stern or impatient, or less friendly. Some reported that they or a colleague raised their voices or shouted at a patient, family member, colleague, or nurse. The following extract refers:

‘Sometimes you do find yourself getting frustrated and find yourself maybe shouting at a patient … I’ll always feel a bit guilty afterwards when you, like, kind of very stern or abrupt to the patient, … you think, like, I probably could have handled that better.’ (P03)

Occasionally, a clinician would become so angry that they would delay or refuse to consult with a patient:

‘But I remember that doctor was really upset, and he took the folder out of the triage. He put it on the side and said, no one is seeing this patient. I’ll see this patient last.’ (P01)

**Sub-theme 1.3: The influence of emotions on work performance:** Distressing emotions affected clinical performance in the workplace. Some participants disclosed that all motivation for their work would cease, and, at times, they would become effectively paralysed by events, not knowing what to do or how to respond. A participant reported:

‘It’s demotivating even to go to hospital, because you know that you’re going to meet this person who is so impatient and harsh and … you don’t want to ask questions, because you don’t know how he’s going to respond …’ (P11)

Occasionally, irritated senior clinicians may criticise, insult, and question junior doctors’ competency. Consequently, after a poor patient outcome event, junior doctors might refrain from asking questions or seeking advice, hesitate to act, doubt their competence, or fail to speak up when they suspect that a senior clinician is making a mistake:

‘So I’m a very timid person … I tend to just go along with what the senior tells me. That’s what I tend to do. I won’t necessarily make it an argument or carry on pushing and asking, but what about this, what about that?’ (P02)

Anxiety affected the ability of interns to perform in the teaching environment, as they became insecure and inept when presenting patients to their seniors. This could affect their due diligence during consultations. The following extract refers:

‘Then I suppose you get people that are frustrated, irritated at the end of the day, then they probably tend to see patients quicker. Don’t really dive into the problem. They always have tunnel vision. It definitely affects how you treat your patients.’ (P01)

#### Theme 2: The emotionally competent doctor

**Sub-theme 2.1: Interns’ inherent emotional management skills:** The participants undoubtedly possessed some emotional management skills and could navigate many challenging situations. Their responses demonstrated that they had insight into why people (patients, family members or other colleagues) were sometimes non-compliant. They spoke of de-escalating the tension and trying to be as supportive as possible, as may be seen in the following extract:

‘And you need to try, like, realise that they’re not being rude to you since you did something wrong. It’s maybe an issue that they’re dealing with … They might be going through something and then they’re just lashing out on you … Just to, like, realise, you know, it’s not you that’s, you know, the problem.’ (P03)

However, some situations exceeded their skill set. The participants acknowledged the need for advanced socio-emotive skills, stating that training in such skills could assist them as clinicians. One participant said:

‘I mean the things … before you are forced to deal with, you need to have your own emotions in check to deal with terrible, terrible things. It’s very easy to sort of, like, crumble in the scenarios that we face … Like, you could easily spiral …, go into a depression … if you are not in the right place.’ (P10)

**Sub-theme 2.2: Specific skills for managing emotions in the workplace:** The participants described the specific skills they regarded as essential for managing emotions: listening to the patient without being talkative; reflecting on personal behaviour; speaking up with confidence; and focusing on the patient’s needs. A participant said:

‘I think definitely more just … learning to listen to patients. … Listen to the patient. And if you don’t interrupt them, and you kind of let them go on with their story, sometimes a lot of your questions will be answered without you having to probe further.’ (P03)

The participants believed that emotional skills improved overall competence, enabling them to fulfil their duties proficiently and reduce patient suffering. In addition, they stated that emotional skills contribute positively to other spheres of the junior doctor’s life, namely, confidence, leadership, teaching abilities, and intimate relationships:

‘It will help us perform better, become better. If we are better emotional versions of ourselves, in everything else we do, we become better. So, I feel like it would help us interact with other healthcare workers … and also it will help … with our patients.’ (P08)

#### Theme 3: The emotional support needs of junior doctors

**Sub-theme 3.1: Sources of emotional support for medical interns:** After a challenging encounter, most participants would seek support from a colleague, friend, or the church. Socialising with friends was useful, as was engaging in physical activity, journaling, or meditating, as may be seen in the following extract:

‘I think one of the big things that helps is talking to friends and colleagues about what you go through. Because I think, you know, especially at our age, we see and deal with things that most people our age don’t – don’t have to deal with … you laugh about the bizarre situations you’ve had to deal with and those crazy things that you see, and I feel like making light … of those situations definitely helps.’ (P03)

**Sub-theme 3.2: Workplace support for medical interns:** A frequently repeated request was for professional emotional support for junior doctors in the workplace, either from a psychologist or a competent senior doctor. Senior doctors need to be encouraged to understand the needs of young doctors better and to discard the idea that a professional doctor will always cope perfectly:

‘If every department has that, but also something tangible that shows that you can be supported …’ (P05)

Participants highlighted the importance of a supportive work environment in which they would feel safe to raise issues:

‘A department that actually teaches these things and normalises, you know, coming forward, talking about problems, making you feel comfortable …’ (P02)

The participants suggested that someone should monitor and identify struggling interns early, instead of waiting for them to request assistance. In addition, they suggested that mental health screening should be available from the first year of medical school. The importance of debriefing after challenging encounters was a recurring request, as may be seen in the following extract:

‘I would just recommend that there’s got to be -– in moments when working in medicine, and you see that things have gone really south – there has got to be debriefing.’ (P01)

### A summary of results

**FIGURE 1:**
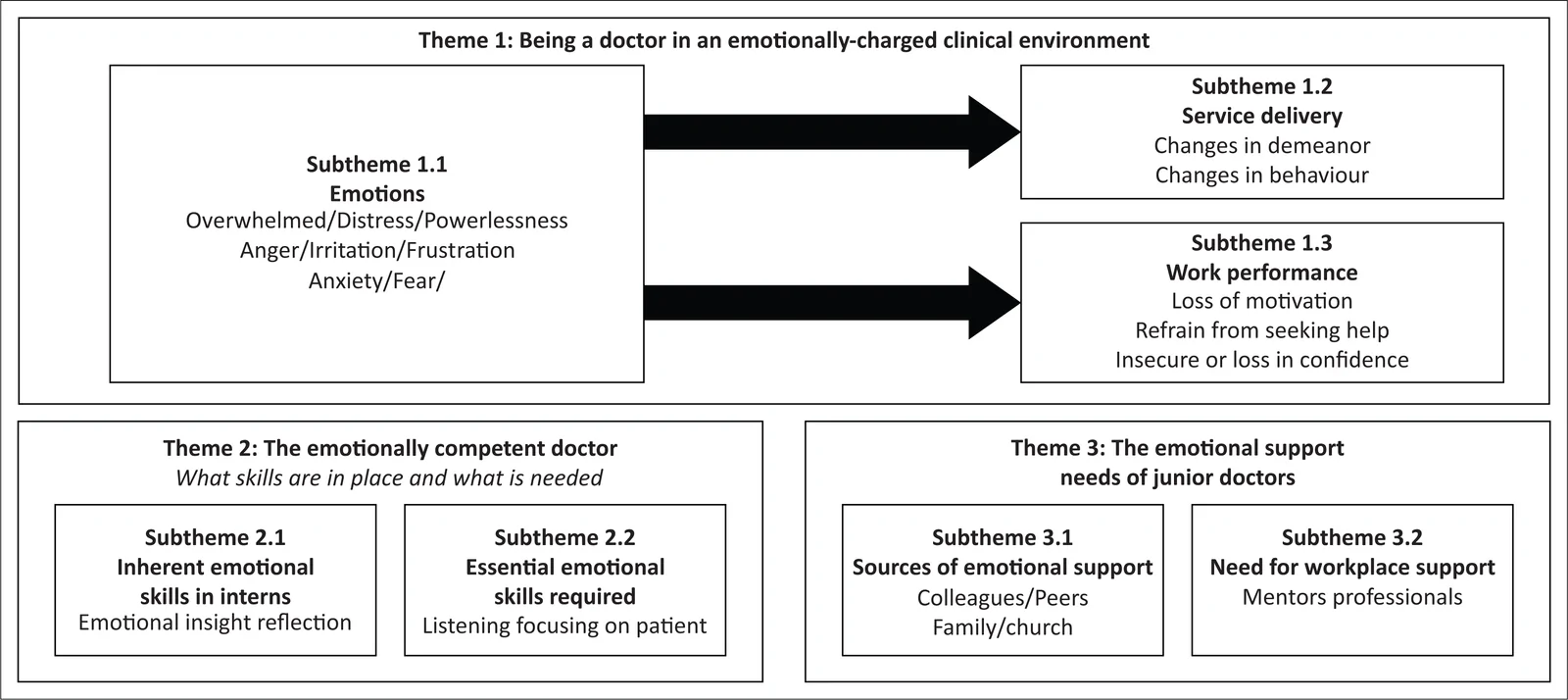
The relationship of themes and sub-themes.

In summary (see Figure 1), the first theme captured the experiences of junior doctors in an emotionally charged clinical environment, with interns reporting experiencing and witnessing a range of difficult emotions, including feelings of overwhelm, stress, irritability, and anxiety. The emotions had a direct impact on behaviour towards patients and subsequent service delivery, as well as negatively affecting work performance and the ability to learn.

The second theme captured the skills junior doctors reported as helping them to manage emotionally charged encounters, while the final theme focused on the support junior doctors need to cope with the emotional challenges they face in the workplace.

## Discussion

This study highlights the importance of regulating strong emotions in the workplace, not only for junior doctors but also for senior clinicians who supervise them. Emotions may play a key role in directing and controlling behaviour,^2^ thereby affecting both service delivery and patients’ clinical outcomes.

Junior doctors described a range of highly charged emotional interactions. All of them evoked powerful and distressing emotions, such as feelings of demotivation, hopelessness, anger, irritation or anxiety, experienced not only by doctors but also by patients and their family members. Emotions are a universal expression of our humanity. Panksepp,^2^ a pioneer in affective neuroscience, identified three powerful negative primal emotions that operate as biological impulses that drive behaviour: *fear, rage*, and *distress/grief*. These are important aspects of the survival system and trigger the fight-or-flight/freeze/fawn response.^2^ The high levels of anxiety the participants described are examples of the *fear* response, the biological impulse to move away from a threat. Irritability and anger are mediated by the neurological pathways of *rage*, the biological impulse to destroy an obstacle in one’s way. Feelings such as demotivation and hopelessness fall under the category of *distress/grief*, the biological impulse to cry out in pain for help.^2^ Emotions, as inner experiences of biological impulses, lead to behaviours that are useful for managing physical threats but can be counterproductive in interpersonal interactions, such as dealing with a colleague or patient who is difficult.^1^

Junior doctors reported counterproductive behaviours following negative emotions, both in themselves and in colleagues, including becoming abrupt, impatient, rude, raising their voice, and even, on occasion, delaying or refusing to see a patient. They described how they became focused on the clinical aspects of the case, potentially missing or ignoring important individual or contextual cues. All of these behaviours were linked to strong emotions and illustrate how, when unregulated, they can lead to specific counterproductive behaviours, such as criticising, preaching, manipulating, or ignoring another person.^1^ Behaviours, such as moving a patient’s file to the bottom of the pile, worsen the situation^1^ and contribute to poor service delivery.^13^

Junior doctors also observed that strong emotions led to behaviours that impacted clinical performance. They reported the negative effect on motivation, and, in some scenarios, a feeling of paralysis occasioned by events to which they felt unable to respond. When junior doctors were exposed to irritable senior clinicians who insulted them and questioned their competence, some were driven inward, to the point of feeling unable to ask questions, seek advice, or point out possible mistakes. These behaviour patterns can contribute to toxic environments, which are strongly linked to negative behaviours such as belittling and humiliation.^21^ Although emotions are powerful drivers of behaviour, they can be modified or regulated.^1^ Higher executive functions enable reflection on emotions and the modification or suppression of instinctive behaviours that may be counterproductive.^22,23^ EC encapsulates the skills needed to understand, become aware of, and regulate emotions and behaviour.^11,12^ It may be assumed that emotional skills develop as doctors mature, but more studies are needed to further characterise how EC evolves over time.

Junior doctors mostly use not only peer support to help them cope but also express a strong need for emotional support from their clinical supervisors, as well as access to workplace professional assistance (employee assistance programme, counsellors or psychologists) within the workplace.

### Study’s limitations

The qualitative nature of the study does not allow for generalisability, but efforts have been made through rich descriptions of the setting to enable the transferability of the findings. Although the four district hospitals through which the interns rotated represented only three districts in one province in South Africa (the Eastern Cape), they are similar to other rural public-sector hospitals in South Africa and the themes that emerged may resonate with clinicians in other parts of the country.

In addition, the final sample included interns from diverse backgrounds and a wide range of universities. Twelve out of the 24 interns who were invited participated, and it is not known whether additional experiences were missed by those who did not participate. There were no significant differences (the hospitals attended, universities, background or gender) between those who agreed and did not agree to participate.

Further studies in other settings within South Africa could expand on interns’ experiences with coping during challenging patient encounters in clinical practice. More studies are needed to further elucidate the findings and quantify the issues and themes in a broader population.

### Recommendations

Doctors are at high risk of burnout and emotional exhaustion.^3,4^ Therefore, the request for clinical supervisors to improve vigilance and awareness of the emotional well-being and mental health of the junior doctors should be taken seriously.^24^ Participants suggested that guided emotional debriefing after stressful workplace interactions could offer much-needed support. This would allow doctors to reflect on events and their emotional impact, especially if they could also witness senior doctors modelling emotionally competent behaviour.^25^ A successful debriefing would require training clinical supervisors in the appropriate skills for leading a debriefing session.^26^

The junior doctors in this study already demonstrated considerable emotional awareness, with all describing EC as an important skill for doctors. Emotional competence, whether gained through training, workplace debriefing or modelling by more senior doctors, could significantly improve service delivery,^13^ improve patient outcomes^5^ and reduce burnout and mental illness among clinicians.^27^ As it is impossible to protect newly qualified doctors from emotionally charged encounters, the authors believe that the development of EC should be included in medical education.^5^ The medical fraternity should also address negative behaviours in the workplace by focusing on creating conducive learning environments.^24^ Further research is needed to identify effective educational activities that may achieve this.

## Conclusion

This study underscores the critical role of EC in the workplace, both for junior doctors and the senior doctors who supervise them. The findings demonstrate how strong emotions, if unregulated, can drive counterproductive behaviours, affecting service delivery and clinical outcomes. Junior doctors need emotional support in the workplace, and modelling EC by clinical supervisors would assist their development. Medical interns need to develop the capacity to recognise, reflect on, and regulate emotion-driven impulses and counterproductive behaviours in medical practice. Educational activities in medical education need to be developed to support the development of EC.
